# Medical Items

**Published:** 1910-05

**Authors:** 


### MEDICAL ITEMS

In malarial conditions a diuretic is not indicated as often as the symptoms suggest, as one always has to contend with a torpid liver, that is throwing a part of its work on the kidneys, meaning double duty for the latter.

In such cases the rational treatment is to use some agent which will stimulate all the excretory organs, dividing the duty of each and causing thorough elimination.

Tongaline either alone or in combination with other agents, as indicated, will invariably expel the malarial and other poisons promptly and thoroughly.

---

The extract of cod liver oil used in the preparation of Hagee's Cordial of the Extract of Cod Liver Oil Compound, is made under such conditions that the medicinally active principles of the oil are separated from the fatty materials without in the least changing their state of combination or solubility, so that even the most complex specific lecithine of cod liver oil is contained as such in the extract and transferred unchanged to the cordial.

Clinical experience with Hagee's Cordial, (an experience which has now extended over many years throughout the Untied States), justifies the assertion that its therapeutic indications are precisely those which belong to cod liver oil in its natural condition.

---

### PAINFUL MONTHLY PERIODS.

Many Doctors prescribe a combination of Dioviburnia and Neurosine (equal parts) to abate the pain and nervousness of Dysmenorrhea. Dioviburnia acting as a reconstructor to the parts affected, Neurosine allaying the pain, resuscitating and toning the nervous system. Physicians can prescribe Diovuburnia and Neurosine with impunity as these products contain no opium, morphine, chloral or other deleterious drugs.

